# Berberine Attenuates Nonalcoholic Hepatic Steatosis by Regulating Lipid Droplet‐Associated Proteins: In Vivo, In Vitro and Molecular Evidence

**DOI:** 10.1111/jcmm.70524

**Published:** 2025-04-07

**Authors:** Hongzhan Wang, Zhi Wang, Dingkun Wang, Kexin Nie, Wenbin Wu, Yang Gao, Shen Chen, Xinyue Jiang, Yueheng Tang, Hao Su, Meilin Hu, Ke Fang, Hui Dong

**Affiliations:** ^1^ Institute of Integrated Traditional Chinese and Western Medicine, Tongji Hospital Tongji Medical College, Huazhong University of Science and Technology Wuhan China; ^2^ Department of Integrated Traditional Chinese and Western Medicine, Tongji Hospital Tongji Medical College, Huazhong University of Science and Technology Wuhan China; ^3^ Department of Rehabilitation Medicine, Tongji Hospital Tongji Medical College, Huazhong University of Science and Technology Wuhan China

**Keywords:** berberine, BSCL2, CIDEA, lipid droplet, NAFLD, perilipins

## Abstract

Hepatic lipid droplet (LD) accumulation is a hallmark of nonalcoholic fatty liver disease (NAFLD). Although the clinical efficacy of berberine (BBR) in treating NAFLD has been established, the mechanism remains uncertain. This study is to evaluate the effects of BBR on hepatic LDs and investigate the underlying mechanisms. Using high‐fat diet‐induced obese (DIO) mice as the model for NAFLD, BBR was administered daily by gavage for 4 weeks. Liver tissue was examined for changes in lipid deposition and histology. Transcriptomics was performed to screen differently expressed genes. The potential targets of BBR against NAFLD were then determined by Western Blot and immunostaining. In oleic acid (OA)‐induced HepG2 cells, the link between BBR and potential targets was further elucidated through the activation or antagonism of PPARα. The binding of BBR to potential targets was predicted using molecular docking. BBR significantly reduced hepatic steatosis by decreasing LD size rather than number. Transcriptomics with validation demonstrated that BBR modulated the expression of LD‐associated proteins CIDEA and PLIN4 in the liver. Further investigations revealed that BBR reversed the abnormal elevation of BSCL2 and PLIN2 in steatotic livers. Finally, we found that BBR reduced LD size in OA‐induced HepG2 cells by regulating BSCL2 and PPARα‐mediated CIDEA/PLIN4/PLIN2. Notably, BBR could bind well to PPARα and BSCL2. BBR can attenuate hepatic steatosis in DIO mice by reducing LD size through the regulation of LD‐associated proteins.

**Abbreviations**
ARF1: adenosine diphosphate‐ribosylation factor 1
ATGL: adipose triglyceride lipase
BSCL: Berardinelli‐Seip congenital lipodystrophy
CFD: complement factor D
CIDEA: cell death‐inducing DNA fragmentation factor alpha‐like effector A
DG: diglyceride
DGAT: diacylglycerol acyltransferase
DIO: diet‐induced obese
ER: endoplasmic reticulum
FABP4: fatty‐acid‐binding protein 4
FFA: free fatty acid
GPAT4: glycerol‐3 phosphate acyltransferase 4
HSL: hormone‐sensitive lipase
LD: lipid droplet
LXRα: Liver X receptor α
MOGAT1: monoacylglycerol acyltransferase 1
NAFLD: nonalcoholic fatty liver disease
OA: oleic acid
PLIN: perilipin
PPAR: peroxisome proliferator‐activated receptor
SCD1: stearoyl‐CoA desaturase 1
SREBP‐1: sterol regulatory element binding protein‐1
TG: triglyceride

## 1 Introduction

Nonalcoholic fatty liver disease (NAFLD) is the most prevalent liver disease worldwide, encompassing a spectrum of conditions ranging from nonalcoholic fatty liver to nonalcoholic steatohepatitis, fibrosis, cirrhosis and ultimately liver cancer. The global prevalence of NAFLD is more than 30% and is increasing year by year [1, 2]. Beyond liver‐related complications, NAFLD increases the risk of cardiovascular disease, which is the most common cause of mortality in individuals with NAFLD [3]. It is also associated with a higher incidence of chronic diseases such as type 2 diabetes mellitus, hypothyroidism, chronic kidney disease and polycystic ovary syndrome [4]. The therapies as intake restriction and lifestyle modification are effective but difficult to maintain due to the lack of patient compliance. Pharmacological treatments, including lipid‐regulating and hepatoprotective agents, offer limited efficacy and may cause adverse effects. Thus, it is urgent to seek new, safe, effective, and highly targeted therapies.

Lipid droplet (LD) is known to be an organelle derived from the endoplasmic reticulum that plays an essential role in lipid storage, transport, and metabolism. The periphery of LD is a phospholipid monolayer inlaid with various proteins, with the interior consisting of neutral lipid cores such as diglycerides (DGs), triglycerides (TGs) and cholesterol esters. The size, number, lipid, and protein composition are highly dynamic, adapting to metabolic changes [5]. Obesity is an important cause of NAFLD and closely related to the severity of NAFLD [6]. In obese individuals, lipids that exceed the storage capacity of adipose tissue are often spilled into the liver to form large amounts of giant LDs. Hepatic LD accumulation is an important hallmark of NAFLD and plays a central role in disease progression [7]. Targeting LD function and regulation in hepatocytes may therefore provide a new perspective in the treatment of NAFLD [8].

Berberine (BBR) is a natural alkaloid as well as one of the main active components of *Coptis chinensis*. BBR has demonstrated beneficial effects in various systemic disorders, including metabolic, cardiovascular, and gastrointestinal diseases. Its diverse biological activities include blood glucose reduction, lipid profile regulation, antioxidant and anti‐inflammatory effects and gut microbiota modulation [9, 10, 11, 12, 13]. The reported mechanisms of BBR in the treatment of NAFLD include improvement of insulin resistance, modulation of lipid metabolism, reduction of oxidative stress and regulation of gut microbiota, among others [14, 15]. Few studies, however, have focused on the connection between BBR and LD, and it remains unclear whether BBR acts on hepatic LD dynamics. Here, we investigated the effect of BBR on hepatic LDs in diet‐induced obese (DIO) mice and explored underlying mechanisms through transcriptomics and in vitro experiments.

## 2 Materials and Methods

### 2.1 Materials and Reagents

The high‐fat diet (D12492) was purchased from Research Diets (USA). BBR (Purity ≥ 98%) was purchased from Shanghai yuanye Bio‐Technology Co. Ltd. (China). The TG Assay Kit was purchased from NanJing JianCheng Bioengineering Institute (China). The Oil Red O Stain Kit was purchased from Biossci (BP037, China). The TruseqTM RNA sample prep kit was obtained from Illumina (USA). The RNA‐easy Isolation Reagent (R701) and HiScript II Q RT SuperMix (R222‐01) were both purchased from Vazyme (China). The oleic acid (OA), GW6471 (a PPARα antagonist) and Fenofibrate (a PPARα agonist) were purchased from SelleckChem (E4087, S2798, S1794, China). The cell counting kit‐8 (CCK8) and PAGE Gel Quick Preparation Kit were purchased from Yeasen Biotechnology Co. Ltd. (China).

The primary antibodies were as follows: Cell death‐inducing DNA fragmentation factor alpha‐like effector A (CIDEA, 13170‐1‐AP, Proteintech), perilipin 4 (PLIN4, NBP2‐13776, Novusbio), stearoyl‐CoA desaturase 1 (SCD1, GB113844, Servicebio), complement factor D (CFD, A8117, Abclonal), fatty‐acid‐binding protein 4 (FABP4, A0232, Abclonal), adenosine diphosphate‐ribosylation factor 1 (ARF1, A9195, Abclonal), Berardinelli‐Seip congenital lipodystrophy 2 (BSCL2, A14583, Abclonal), PLIN2 (A6276, Abclonal), PLIN5 (A20418, Abclonal), glycerol‐3‐phosphate acyltransferase 4 (GPAT4, A20804, Abclonal), diacylglycerol acyltransferase 1 (DGAT1, A6857, Abclonal), DGAT2 (A13891, Abclonal), liver X receptor α (LXRα, A3974, Abclonal), sterol regulatory element binding protein‐1 (SREBP‐1, GB113804‐100, Servicebio), peroxisome proliferator‐activated receptor α (PPARα, 15,540‐1‐AP, Proteintech), PPARγ (A11183, Abclonal), adipose triglyceride lipase (ATGL, A5126, Abclonal), hormone‐sensitive lipase (HSL, A24689, Abclonal), glyceraldehyde‐3‐phosphate dehydrogenase (GAPDH, AC002, Abclonal;60,004‐1‐Ig, Proteintech). The secondary antibodies included Anti‐rabbit IgG (H + L) Dylight 800 (5151, CST), Anti‐mouse IgG (H + L) Dylight 680 (5470, CST), HRP‐conjugated Goat Anti‐Rabbit IgG (GB23303, Servicebio), Goat Anti‐Rabbit IgG Dylight 594 (A23420, Abbkine). The rest of the unmentioned reagents were purchased from Servicebio Technology Co. Ltd. (China).

### 2.2 DIO Model and Animal Treatment

The animal experiments were approved by the Institutional Animal Care and Use Committee at Huazhong University of Science and Technology, Wuhan, China (No. 2915). All male C57BL6/J mice (7 weeks old) were obtained from Charles River and housed in an SPF‐grade animal laboratory (12 h dark/light cycles, 20°C–23°C and 55%–65% humidity). All mice were provided with unlimited access to food and water. After 1 week of adaptation, mice were randomly assigned to either the normal diet group (N, *n* = 8) or the DIO group (*n* = 24), which received a 60% kcal high‐fat diet. After 12 weeks, DIO mice were randomised into the model group (M), low‐dose BBR group (L) and high‐dose BBR group (H), with 8 mice per group. The doses of BBR were determined based on previous reports [16, 17]. The protocol for daily gavage in the four groups was as follows: Group N (normal saline), group M (normal saline), groups L and H (150 mg/kg and 300 mg/kg of BBR). The intervention lasted 4 weeks. Subsequently, all mice were anaesthetised and executed. Fresh liver tissues were harvested for transmission electron microscopy sample preparation, 4% paraformaldehyde fixation (for paraffin sections or frozen sections) and freezing at −80°C, respectively.

### 2.3 HE Staining

Xylene and gradient ethanol were used to dewax the paraffin sections of liver. After that, the sections were stained with haematoxylin and eosin respectively. Images were taken using a biomicroscope (BX‐53, Olympus).

### 2.4 Determination of TG Content

After homogenisation of liver tissue or ultrasonic breaking of cells, TG concentration was measured utilising the TG Assay Kit according to the kit instructions. TG content was obtained through dividing the TG concentration by the corresponding protein concentration.

### 2.5 Oil Red O Staining

For Oil Red O staining of liver tissue, frozen sections were dried at room temperature before being stained with Oil Red O working solution. Nuclei were lightly stained with haematoxylin and finally rinsed and mounted. Imaging was conducted with a biomicroscope (CX‐31, Olympus). ImageJ (v1.52a) was used to measure the stained area in each group under an 800X magnification field. For staining of cells, cells were fixed with paraformaldehyde for 10 min and then stained with Oil Red O working solution for 10 min.

### 2.6 Transmission Electron Microscopy

After the mice were anaesthetised, fresh liver tissue was taken quickly and immersed in 2.5% glutaraldehyde. After ultrathin sectioning and staining of tissue embedded in epoxy resin, imaging was performed using a transmission electron microscope (HT7800, HITACHI). ImageJ (v1.52a) was used to measure the size and number of LDs.

### 2.7 Transcriptomics

Total liver RNA was obtained from mice in groups N, M and H using the Trizol method. The concentration, purity and integrity of RNA were assayed separately, followed by detection of RIN value (Agilent2100). mRNA was isolated using the magnetic rack. The mRNA‐seq library was created by TruseqTM RNA sample prep kit, and then quantified by Quantus Fluorometer (Promega). Then, sequencing was conducted by NovaSeq 6000 (Illumina). Mapped data (reads) were obtained by comparing it to a reference genome. The expression levels of genes and transcripts were quantitatively analysed utilising the software RSEM and differently expressed genes (DEGs) among samples were screened by DESeq2 software. KEGG and GO enrichment analyses were performed by KOBAS and Goatools, respectively. Protein–Protein Interaction Networks (PPI) analysis was conducted for DEGs in groups N, M and H by STRING database.

### 2.8 Reverse Transcription‐Quantitative Polymerase Chain Reaction (RT‐qPCR)

Total RNA was extracted from liver tissue using RNA‐easy Isolation Reagent after homogenisation. RNA purity was assessed by OD260/280, with an acceptable range of 1.8–2.2. The concentration of RNA should be adjusted to 0.8–1.2 μg/μL. RNA was then reverse transcribed by using HiScript II Q RT SuperMix, followed by RT‐qPCR (A48141, Thermo Fisher Scientific). The quantification of mRNA was performed according to the formula: 2^−ΔΔCT^. All Primer sequences involved in this study are shown in Table 1 (Table 1).

**TABLE 1:** Primer sequences used for RT‐qPCR.

Gene	Forward	Reverse
Cidea	TGACATTCATGGGATTGCAGAC	CATGGTTTGAAACTCGAAAAGGG
Plin4	GTGTCCACCAACTCACAGATG	GGACCATTCCTTTTGCAGCAT
Scd1	TTCTTGCGATACACTCTGGTGC	CGGGATTGAATGTTCTTGTCGT
Cfd	TACATGGCTTCCGTGCAAGTG	CACAGAGTCGTCATCCGTCA
Fabp4	AAGGTGAAGAGCATCATAACCCT	TCACGCCTTTCATAACACATTCC
Mogat1	TGGTGCCAGTTTGGTTCCAG	TGCTCTGAGGTCGGGTTCA
Gapdh	AGGTCGGTGTGAACGGATTTG	TGTAGACCATGTAGTTGAGGTCA

### 2.9 Western Blot

Total liver protein was extracted by homogenising tissue in protein lysis buffer, while HepG2 cell protein was extracted by ultrasonic disruption in the same buffer. The concentration of protein was determined by the BCA reagent. After protein electrophoresis, transmembrane electrophoresis was conducted with PVDF membranes. Next, the membranes were blocked with 5% skim milk and placed in a primary antibody incubation kit for overnight shaking at 4°C. Imaging was implemented by the Odyssey infrared imaging system (Li‐Cor Biosciences) after incubation with secondary antibodies.

### 2.10 Immunostaining

For immunohistochemistry (IHC), the paraffin sections were dewaxed with xylene and a graded ethanol series, followed by antigen repair and endogenous peroxidase blocking. Non‐specific antigens were blocked with goat serum. Next, the sections were incubated with primary antibodies (diluted at 1:200) and secondary antibodies (diluted at 1:500), respectively. After diaminobenzidine staining, nuclei were stained using haematoxylin. Images were captured using a biomicroscope (BX‐53, Olympus). Mean optical density (MOD) was quantified by image J (1.52a).

For immunofluorescence (IF) of paraffin sections, serum blocking was conducted directly after antigen repair. The sections were incubated with specific antibodies, then stained with DAPI before mounting. For cellular IF, cells were fixed with 4% paraformaldehyde for 10 min, then incubated with 0.5% Triton X‐100 for 5 min, followed by the same steps as above. Images were captured using a biomicroscope (BX‐53, Olympus). Mean fluorescence intensity (MFI) was quantified using image J (1.52a).

### 2.11 Cell Culture and Intervention

HepG2 cells, obtained from Pricella Life Science &Technology Co. Ltd. (CL‐0103), were used to preliminarily explore the regulatory mechanisms of BBR on hepatic LD‐associated proteins. Cells were cultured at 5% CO_2_ and 37°C using the supplier‐recommended medium (Minimum Essential Medium, 10% fetal bovine serum, 1% Penicillin–Streptomycin Solution). Passaging was performed when the cells grew to 80% fusion. Before intervention, cells were starved in serum‐free medium for 6 h. They were then stimulated for 24 h with OA solution alone or OA solution pre‐added with BBR, GW6471 + BBR, or Fenofibrate. Cells cultured in serum‐containing medium were served as the control (Con). The concentration of OA was determined based on previous reports and CCK8 results [18]. The concentration of BBR was determined based on CCK8 and therapeutic efficacy. The concentrations of GW6471 and Fenofibrate were referenced to previous studies [19, 20]. The cells after intervention were used for subsequent experiments.

### 2.12 CCK8 Test

Cells were stimulated for 24 h with OA solution (0.5 mM, 1 mM and 2 mM) or BBR (5 μM, 10 μM, 20 μM, 30 μM, 40 μM, 60 μM), respectively. Subsequently, the CCK8 test was performed and cell viability was analysed according to the manufacturer's instruction. After determining the OA concentration, cells were stimulated using OA solution with or without BBR added for 24 h, and cell viability was determined.

### 2.13 Molecular Docking

The chemical structure of BBR was extracted from the PubChem database. The protein structures of PPARα (2ZNN) and BSCL2 (6MLU) were obtained from the PDB database. Molecular docking was performed using AutoDock (v1.5.7), and visualisation was performed using PyMOL (v2.2.0).

### 2.14 Statistical Analysis

The data were analysed by GraphPad Prism (8.0.2) and were presented as mean ± standard deviation. Statistical significance was assessed by one‐way analysis of variance, Dunnett's *t* test and Dunnet's T3 test. *p* < 0.05 was assumed to be statistically significant.

## 3 Results

### 3.1 BBR Attenuates Hepatic Steatosis and Reduces LD Size in the Liver of DIO Mice

**FIGURE 1:**
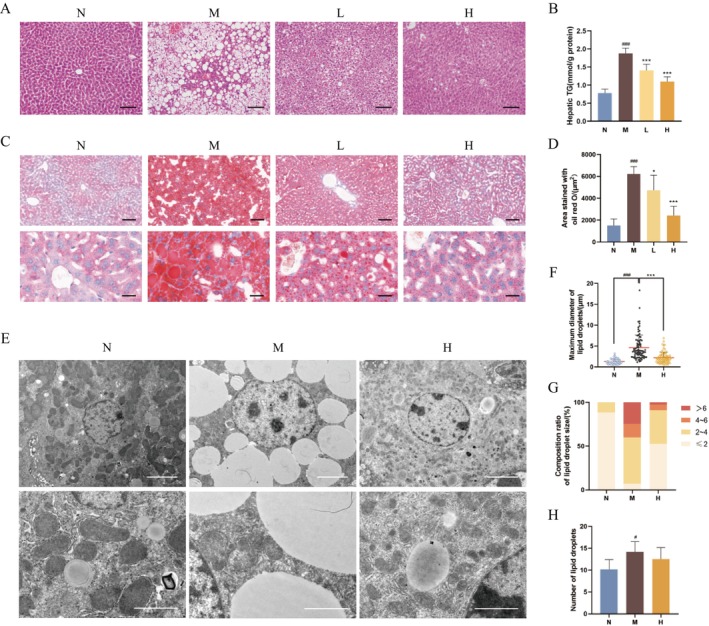
BBR reduces LD size and attenuates hepatic steatosis in the liver of DIO mice. (A) HE images of mouse livers. Scale bar: 100 μm (*n* = 6/group). (B) Hepatic TG content in each group (*n* = 5/group). (C,D) Oil Red O imaging of liver and statistics of stained area. Scale bars: 100 μm and 25 μm, respectively (*n* = 6/group). (E) Representative transmission electron microscopy images of liver in the N, M and H groups. Scale bars: 5 μm and 2 μm, respectively (20–25 LDs/mouse, *n* = 3/group). (F) Quantification of the maximum diameter of LDs (each point represents one LD. 3 fields of view were taken for each sample). (G) Composition ratio of LD size. (H) The number of LDs in each field of view. ###*p* < 0.001, #*p* < 0.001 compared with the N group; *p < 0.05, ***p < 0.001 compared with the M group.

Compared to normal mice, DIO mice showed a noticeable increase in hepatic TG content and large quantities of LD vacuoles of varying sizes in the liver, confirming the successful modelling of a mice model of NAFLD. BBR treatment attenuated these abnormal changes, with high‐dose BBR demonstrating the most pronounced effects (Figure 1A,B). Oil Red O staining further supported these findings, showing a greater accumulation of round or quasi‐round red LDs in the liver of DIO mice, which was differentially ameliorated by BBR treatment (Figure 1C,D). Based on its superior efficacy in reducing hepatic steatosis, high‐dose BBR was selected for subsequent studies.

The liver tissues of mice in the N, M and H groups were selected for transmission electron microscopy imaging (Figure 1E). More and larger LDs were produced in the cytoplasm of the liver with NAFLD, even some of which showed fusion. BBR treatment significantly reversed the morphology changes of LDs described above. Quantitative analysis showed that high‐dose BBR could decrease the proportion of large‐diameter LDs (d > 4 μm) and increase the proportion of small‐diameter LDs (d ≤ 4 μm) in the liver of DIO mice without affecting the overall LD number, indicating that BBR attenuates hepatic steatosis by reducing LD size (Figure 1F–H).

### 3.2 Transcriptomics Reveals DEGs Associated With the Effect of BBR in Steatotic Liver

**FIGURE 2:**
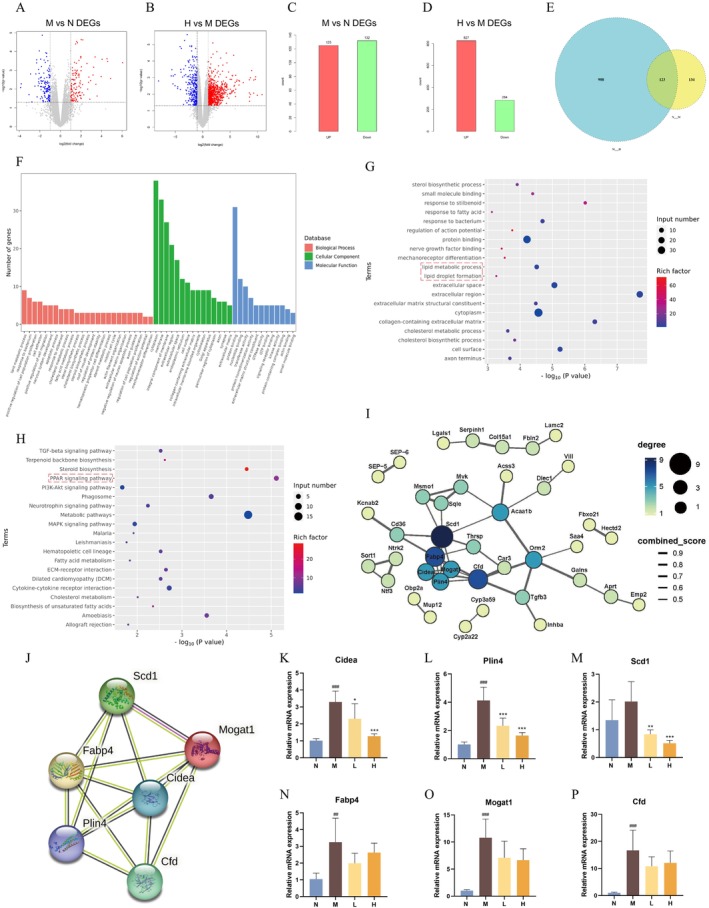
Transcriptomics of mouse liver from the N, M and H groups. (A,C) Volcano plot and quantitative statistics of DEGs between the M and N groups. (B,D) Volcano plot and quantitative statistics of DEGs between the H and M groups. (E) Statistics on the overlapping DEGs among the three groups. (F) GO functional classification for the overlapping DEGs. (G) GO enrichment analysis for the overlapping DEGs. (H) KEGG enrichment analysis for the overlapping DEGs (*n* = 3/group). (I) PPI analysis for the overlapping DEGs. (J) PPI analysis for the key overlapping DEGs. (K‐P) The mRNA expression of the key overlapping DEGs, *Cidea*, *Plin4*, *Scd1*, *Fabp4*, *Mogat1*, *Cfd* (*n* = 5/group). ###*p* < 0.001 compared with the N group; ***p < 0.001 compared with the M group.

To explore the potential pharmacological mechanisms of BBR on hepatic LDs, liver tissues were analysed by transcriptomics. In DIO mice, 125 DEGs were upregulated, while 132 DEGs were downregulated. Following BBR treatment, 827 DEGs were upregulated and 284 DEGs were downregulated (Figure 2A–D). Then, 123 overlapping DEGs were screened to identify potential targets of BBR for treating NAFLD (Figure 2E, Table S1). GO functional classification showed that lipid metabolic processes, cytoplasm and protein binding were at the top of each category, respectively (Figure 2F). KEGG enrichment analysis revealed a high significance of the PPAR signalling pathway. Notably, GO enrichment analysis revealed two terms, LD formation and lipid metabolic process, indicating a novel perspective to study the efficacy of BBR on regulating hepatic LDs (Figure 2G,H).

According to the results of PPI, the proteins encoded by *Scd1*, monoacylglycerol acyltransferase 1 (*Mogat1*), *Cidea*, *Fabp4*, *Plin4* and *Cfd* presented a dense network of interactions with high connectivity and binding scores (Figure 2I,J). We therefore selected these six genes for further validation. RT‐qPCR showed that the mRNA expression levels of *Cidea* and *Plin4* were all significantly elevated in the liver of DIO mice but decreased after high‐dose BBR treatment. Despite the elevated expression levels of *Mogat1*, *Fabp4* and *Cfd*, they did not appear to be significantly reversed by high‐dose berberine. Additionally, hepatic *Scd1* in DIO mice increased but was not statistically significant compared to the normal diet‐fed mice (Figure 2K–P). The inconsistencies observed between the RT‐qPCR and transcriptomics results may be attributed, at least in part, to the within‐group differences. Therefore, the subsequent studies will be performed to validate these genes at the protein level.

### 3.3 LD‐Associated Proteins CIDEA and PLIN4 Mediate the Regulation of BBR on Hepatic LD

**FIGURE 3:**
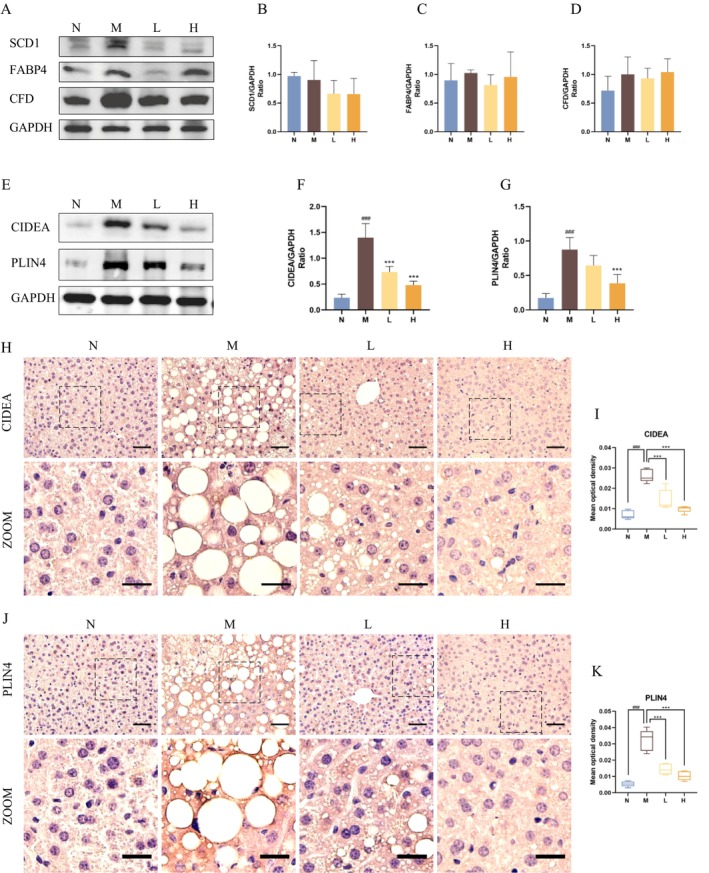
LD‐associated proteins CIDEA and PLIN4 mediate the regulation of BBR on hepatic LD. (A–D) Western blot images of SCD1, FABP4 and CFD and corresponding statistical analysis (*n* = 4/group). (E–G) Western blot images of CIDEA and corresponding statistical analysis (*n* = 4/group). (H,I) Representative IHC images of CIDEA and statistical analysis of MOD. Scale bars: 50 μm and 25 μm (*n* = 4/group). (J,K) Representative IHC images of PLIN4 and statistical analysis of MOD. Scale bars: 50 μm and 25 μm (*n* = 4/group). ###*p* < 0.001 compared with the N group; ***p < 0.001 compared with the M group.

Consistent with RT‐qPCR results, no significant differences were found in the protein expression levels of SCD1, FABP4 and CFD among the four groups (Figure 3A–D). We noted that high‐dose BBR effectively suppressed the overexpression of CIDEA and PLIN4 in the liver of DIO mice (Figure 3E–G). We therefore focused our attention on LD‐associated proteins, CIDEA and PLIN4. Next, immunostaining revealed that CIDEA and PLIN4 were highly enriched around LDs in the liver of DIO mice but were markedly reduced in BBR‐treated mice (Figure 3H–K). On the basis of these findings, LD‐associated proteins CIDEA and PLIN4 may be the potential regulatory targets of BBR.

### 3.4 BBR Regulates LD‐Associated Proteins BSCL2 and PLIN2 in the Liver of DIO Mice

**FIGURE 4:**
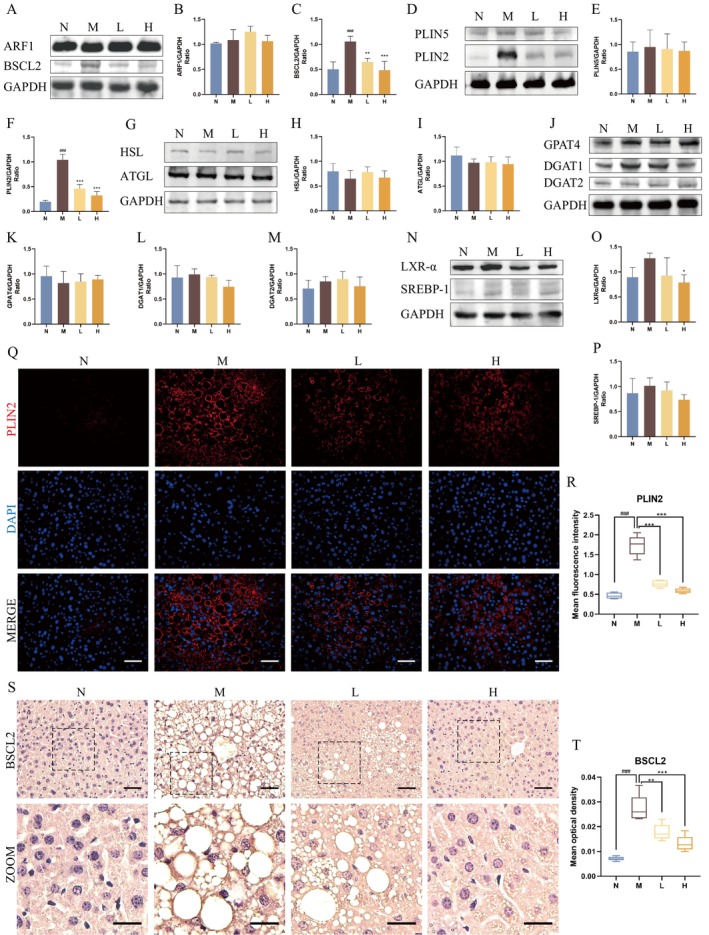
BBR regulates LD‐associated proteins BSCL2 and PLIN2 in the liver of DIO mice. (A–C) Western blot images of ARF1, BSCL2 and corresponding statistical analysis (*n* = 4/group). (D–F) Western blot images of PLIN5, PLIN2 and corresponding statistical analysis (*n* = 4/group). (G–I) Western blot images of HSL, ATGL and corresponding statistical analysis (*n* = 4/group). (J–M) Western blot images of GPAT4, DGAT1, DGAT2 and corresponding statistical analysis (*n* = 4/group). (N–P) Western blot images of LXR‐α, SREBP‐1 and corresponding statistical analysis (*n* = 4/group). (Q,R) Representative IF images of PLIN2 and statistical analysis of MFI. Scale bar: 50 μm (*n* = 5/group). (S,T) Representative IHC images of BSCL2 and statistical analysis of MOD. Scale bars: 50 μm and 25 μm (*n* = 5/group). ###*p* < 0.001 compared with the N group; *p < 0.05, **p < 0.01 and ***p < 0.001 compared with the M group.

To investigate the effects of BBR on LD‐associated proteins in the liver, we analysed key regulators of LD biogenesis and expansion (ARF1, BSCL2), LD lipolysis (PLIN2, PLIN5, ATGL and HSL) and LD growth (GPAT4, DGAT1 and DGAT2) (Figure 4A–M). We also detected the key molecules (LXRα, SREBP‐1) that regulate the de novo fatty acid synthesis pathway (Figure 4N–P). No significant differences were found in ARF1, PLIN5, GPAT4, DGAT1, DGAT2 among the groups, while the expression of BSCL2 and PLIN2 was notably elevated in DIO mice and reduced following BBR treatment. ATGL and HSL showed a slight decrease in DIO mice, while LXRα and SREBP‐1 were slightly increased, but these changes were not statistically significant. Similar changes in the expression levels of BSCL2 and PLIN2 were also observed by immunostaining (Figure 4Q–T). These findings suggested that BBR‐mediated regulation of hepatic LDs is related to the suppression of BSCL2 and PLIN2.

### 3.5 BBR Reduces LD Size in OA‐Induced HepG2 Cells by Regulating PPARα

**FIGURE 5:**
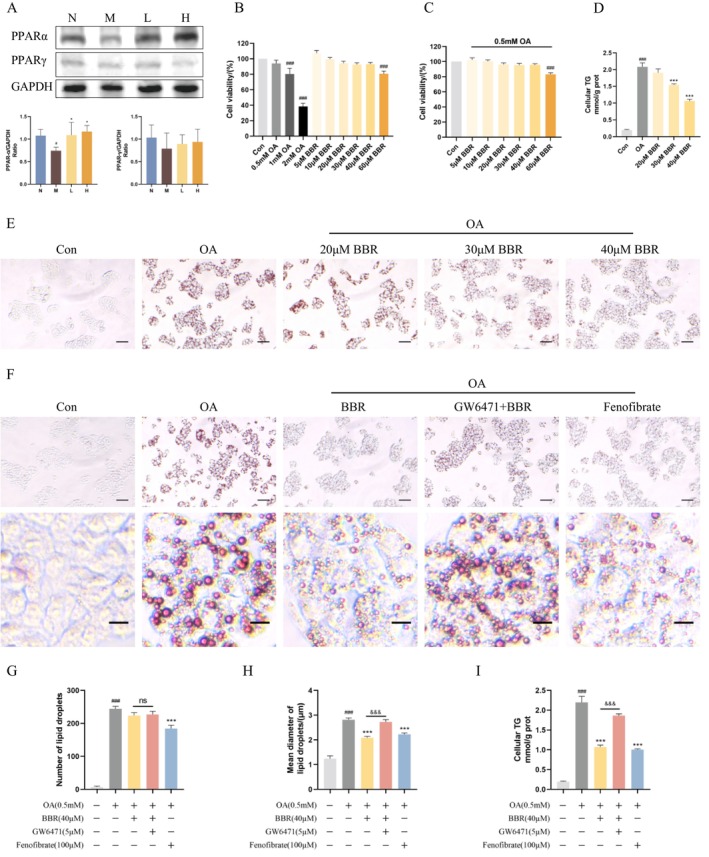
BBR reduces LD size in OA‐induced HepG2 cells by regulating PPARα. (A) Protein expression levels of PPARα and PPARγ in the livers of mice in each group (*n* = 4/group). #*p* < 0.05 compared with the N group; *p < 0.001 compared with the M group. (B) CCK8 test of OA and BBR. (C) CCK8 test after simultaneous intervention of OA and BBR. (D) Cellular TG content after treatment with BBR (20 μM, 30 μM, 40 μM). (E) Oil Red O staining of OA‐induced HepG2 cells after treatment with BBR (20 μM, 30 μM, 40 μM). Scale bar: 50 μm (*n* = 3/group). (F–H) Oil Red O staining of OA‐induced HepG2 cells after treatment with BBR, GW6471 + BBR and Fenofibrate. And Statistical analysis of the number and mean diameter of LDs. Scale bars: 50 μm and 10 μm (*n* = 3/group). (I) Cellular TG content after treatment with BBR, GW6471 + BBR and Fenofibrate (*n* = 3/group). ###*p* < 0.05 compared with the Con group; ***p < 0.001 compared with the OA group; &&&*p* < 0.001 compared with the BBR group; ns, No statistical significance.

Based on the high significance of the PPAR signalling pathway in KEGG analysis, we sought to examine the expression of PPARα and PPARγ. BBR could reverse the condition of low expression of PPARα in DIO mice but not significantly affect the expression of PPARγ (Figure 5A). In vitro, we therefore used OA‐induced HepG2 cells to further investigate the connections between BBR, PPARα and hepatic LD‐associated proteins. Cell viability was significantly inhibited at 1 mM and 2 mM of OA, as well as at 60 μM of BBR (Figure 5B,C). Therefore, 0.5 mM OA and 20 μM, 30 μM and 40 μM BBR were used to observe the effect of BBR on OA‐induced HepG2 cells. In OA‐induced HepG2 cells, BBR at 20 μM, 30 μM and 40 μM reducted cellular TG content and alleviated lipid deposition to varying degrees, with the most pronounced effect at 40 μM (Figure 5D,E). We therefore selected 40 μM BBR for subsequent experiments. Both BBR and Fenofibrate, a PPARα agonist, were able to reverse OA‐induced abnormal LD accumulation and reduce cellular TG content. Unlike BBR, which reduced LD size without significantly decreasing LD number, Fenofibrate was able to affect both the size and number of LDs. GW6471, a PPARα antagonist, markedly blocked the above effects of BBR (Figure 5F–I). Based on these results, it is inferred that PPARα mediates the effect of BBR on LD size in OA‐induced HepG2 cells.

### 3.6 Modulation of CIDEA, PLIN2 and PLIN4 by BBR Depends on PPARα, Contrasting With the Unaffected Regulation of BSCL2 by BBR

**FIGURE 6:**
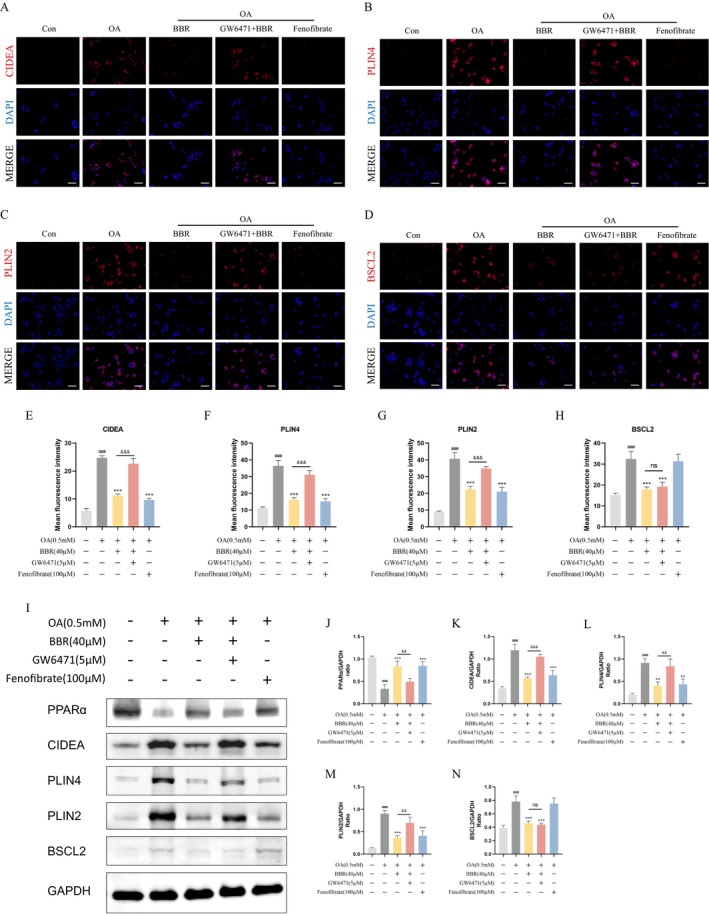
The modulation of CIDEA, PLIN2, and PLIN4 by BBR depends on PPARα, contrasting with the unaffected regulation of BSCL2 by PPARα. (A,E) Representative IF images of CIDEA and statistical analysis of MFI. Scale bar: 50 μm (*n* = 3/group). (B,F) Representative IF images of PLIN4 and statistical analysis of MFI. Scale bar: 50 μm (*n* = 3/group). (C,G) Representative IF images of PLIN2 and statistical analysis of MFI. Scale bar: 50 μm (*n* = 3/group). (D,H) Representative IF images of PLIN4 and statistical analysis of MFI. Scale bar: 50 μm (*n* = 3/group). (I–N) Western blot images of PPARα, CIDEA, PLIN4, PLIN2 in HepG2 cells and corresponding statistical analysis (*n* = 3/group). ###*p* < 0.05 compared with the Con group; **p < 0.01 and ***p < 0.001 compared with the OA group; &&*p* < 0.01 and &&&*p* < 0.001 compared with the BBR group; ns, No statistical significance.

**FIGURE 7:**
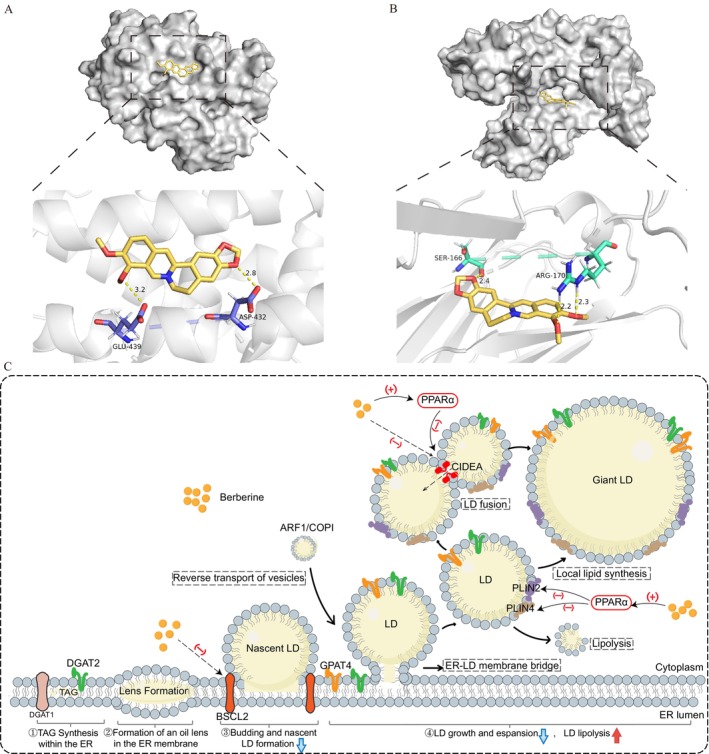
Mechanism of BBR in modulating hepatic LD size in DIO mice. (A) Molecular docking between BBR and PPARα. (B) Molecular docking between BBR and BSCL2. (C) The pattern diagram of BBR reducing hepatic LD size by regulating LD‐associated proteins. Downward arrows represent inhibition, upward arrows represent promotion. (−) Represents the down‐regulation of LD‐associated protein. (+) Represents the up‐regulation of LD‐associated protein.

To determine whether BBR regulates LD‐associated proteins through PPARα, we analysed the expression of CIDEA, PLIN4, PLIN2 and BSCL2 in OA‐induced HepG2 cells. Both BBR and Fenofibrate reversed OA‐induced overexpression of CIDEA, PLIN4 and PLIN2 in HepG2 cells, while the regulatory effect of BBR was notably blocked by GW6471. Interestingly, the abnormal elevation of BSCL2 in OA‐induced HepG2 cells was reversed by BBR but not Fenofibrate, and this effect was not affected by GW6471 (Figure 6A–H). The results of western blot were consistent with the findings above (Figure 6I–N). These findings suggest that the regulation of LD‐associated proteins CIDEA, PLIN4 and PLIN2 by BBR is dependent on PPARα, whereas its effect on BSCL2 is PPARα‐independent. Furthermore, molecular docking revealed that BBR binds stably to both PPARα and BSCL2, with binding energies of −5.35 kcal/mol and − 5.82 kcal/mol, respectively. BBR can form hydrogen bonds at the ASP432 and GLU439 sites of PPARα and at the ARG‐170 and SER‐166 sites of BSCL2 (Figure 7A,B). This implies that BBR is possible to form direct interactions with PPARα and BSCL2.

## 4 Discussion

In this study, we investigated the effect of BBR on LD and LD‐associated proteins both in vitro and in vivo. We found that BBR reduced the size but not the number of LDs in the liver of DIO mice and OA‐induced HepG2 cells. The possible mechanisms include: 1. inhibition of BSCL2‐mediated LD budding and growth; 2. PPARα‐mediated regulation of LD fusion and lipolysis through CIDEA/PLIN4/PLIN2 (Figure 7B).

DIO mice presented a typical pathological feature of hepatic steatosis. BBR significantly reduced hepatic lipid accumulation, which was consistent with previous studies [21, 22, 23]. BBR at high doses greatly reduced the maximum diameter of LDs rather than the number. This result is different from the previous study which showed that BBR also reduced LD number in the liver of DIO mice [22]. However, BBR reduced the size of hepatic LDs in db/db mice and ApoE−/− mice fed a western‐style diet [24, 25]. These inconsistent findings may be attributed to differences in animal models, induction methods and BBR treatment duration.

The effect of BBR on hepatic LDs was further determined by using transcriptomics. Surprisingly, we found that BBR reversed the overexpression of CIDEA and PLIN4 in the liver of DIO mice, but did not significantly affect the protein expression levels of SCD1, FABP4 and CFD. Interestingly, CIDEA and PLIN4 are both LD‐associated proteins, inspiring us to draw a connection between BBR, LD homeostasis and LD‐associated proteins.

Generally, LD biogenesis follows four steps: 1. Free fatty acid (FFA) activation to acyl‐CoA, involved in TAG biosynthesis in the endoplasmic reticulum (ER); 2. nucleation/lens formation of neutral lipids in the ER bilayer; 3. droplets budding in the ER and formation of nascent LDs; 4. recruitment of specific proteins followed by LD growth and expansion through local TAG synthesis and coalescence [26, 27]. The specific proteins recruited in step 4 are divided into two categories: Class I proteins inserted on the ER surface and transported to the LD surface through ER‐LD membrane bridge, e.g., GPAT4, DGAT2; class II proteins targeted to the LD surface from the cytoplasmic lysis through specific structural domains, e.g., CTP: phosphocholine cytidylyltransferase 1 (CCT1), PLINs [28].

CIDEA, a member of the CIDE family, is enriched at the LD‐LD contact and regulates LD fusion [29]. CIDEA is expressed in adipose tissue and the liver of DIO animals [30]. In the present study, high‐dose BBR significantly inhibited the expression of CIDEA around hepatic LDs. PLIN4, a member of the PLIN family, has an 11‐polymer repeating sequence that facilitates its targeting to the LD surface and protects LDs from lipolysis [31]. In our study, BBR inhibited the expression of PLIN4 and weakened the ‘peripheral defence’ ability of LDs, thereby promoting LD lipolysis. It may be a potential mechanism for BBR to regulate LD homeostasis.

We were interested in determining whether additional LD‐associated proteins were involved in BBR‐mediated regulation of LD size. Thus, we evaluated the effect of BBR on several critical LD‐associated proteins involved in LD biological processes. ARF1, a small Ras‐like GTPase, assembles in a complex with coat protein complex I (COPI). They control the formation of the ER‐LD membrane bridge and transport specific TG synthases to LD surface, leading to LD expansion [32, 33]. BSCL2 is an endoplasmic reticulum protein involved in the assembly of LD in the cytoplasm [34]. BSCL2 concentrates at ER‐LD contact, remodels ER membrane to accumulate TG and DG within its unconventional cyclic oligomeric structure, priming LD biogenesis [35, 36]. BSCL2 also provides sites within the ER to promote the formation of ER‐LD membrane bridges, thereby facilitating the growth and expansion of LD [37]. PLIN2, also known as adipose differentiation‐related protein (ADRP), prevents endogenous lipases (e.g., ATGL, HSL) from binding to TAG substrates in LDs, thereby attenuating lipolysis [38]. In this study, we did not confirm the effect of BBR on ARF1, PLIN5, ATGL or HSL, but observed that BBR reversed the aberrant expression of BSCL2 and PLIN2 in steatotic livers. These findings have not been previously reported, but some studies have claimed that BBR regulates the mRNA expression level of PLIN1 in adipocytes [39, 40].

We also examined the expression of key enzymes in the triacylglycerol (TAG) synthesis pathway, such as GPAT4, DGAT1 and DGAT2. All of them are LD‐associated proteins involved in LD biosynthesis and growth [41]. GPAT4 and DGAT2 are transported to the surface of LD through the ER‐LD membrane bridge and promote local lipid synthesis of LD. However, BBR was found to exert no effect on GPAT4, DGAT1 and DGAT2 expression in the present study. Furthermore, given the inclusion of fatty acid metabolism in the KEGG enrichment analysis, we examined the expression levels of the LXRα/SREBP‐1 pathway, which regulates the de novo fatty acid synthesis [42]. Previous studies have demonstrated that BBR inhibits SREBP‐1 expression in ob/ob mice, 3 T3‐L1 adipocytes and rat primary hepatocytes [21, 43, 44]. BBR also induces nuclear translocation of LXRα without altering its expression [45]. However, despite the potential for LXRα inhibition by high‐dose BBR, no aberrant activation of the LXRα/SREBP‐1 pathway was observed in the livers of DIO mice. These inconsistent results may stem from differences in animal models and intervention strategies.

To further investigate how BBR regulates the LD‐associated proteins above, we examined its potential involvement in the PPAR signalling pathway, which was highly enriched in the KEGG enrichment analysis. BBR has been reported to activate PPARγ and exert weight loss, anti‐inflammatory, antioxidant and lipid‐regulating effects [46, 47], while several studies have identified BBR as a PPARα activator [48, 49]. We thus hypothesised that PPAR may mediate, at least in part, the regulation of LD‐associated proteins by BBR. In our study, however, BBR could promote the expression of PPARα with no significant effect on PPARγ in the liver. It is generally known that PPARα is a key regulator of the fatty acid β‐oxidation pathway [50]. In vitro, we found that BBR significantly increased PPARα expression and reduced LD size. This effect may be related to the inhibition of CIDEA‐mediated LD fusion, the promotion of PLIN4/PLIN2‐mediated LD lipolysis, and the inhibition of BSCL2‐mediated LD budding and expansion. BBR blocks the formation of giant LDs to prevent FFAs from causing lipid deposition in hepatocytes, promoting more FFAs to participate in β‐oxidation for energy metabolism, thus ameliorating hepatic steatosis. Although we did not test β‐oxidation‐related genes in this study, prior research strongly supports BBR's role in enhancing fatty acid β‐oxidation [23, 49, 51].

Molecular docking predicts that BBR binds well to PPARα. Recently, Yang et al. reported that BBR regulates the expression of PPARα and PPARγ by targeting AKR1B10 [52]. Therefore, it remains to be further explored whether BBR directly interacts with PPARα to promote its expression. While both our study and Yang et al.'s research examine BBR's role in NAFLD, the focuses differ. Yang et al. identified AKR1B10 as an upstream target of PPAR and observed that berberine modulates fatty acid β‐oxidation and gluconeogenesis downstream of the PPAR signalling pathway through targeting AKR1B10. However, our study aimed to investigate whether BBR affects hepatic LDs through LD‐associated proteins and whether the regulation of these proteins by BBR is mediated by PPARα. We demonstrated that the regulation of CIDEA, PLIN4 and PLIN2 by BBR is dependent on the promotion of PPARα, whereas the regulation of BSCL2 may result from a direct interaction of BBR. It was reported that PLIN2 and PLIN5 are primarily regulated by PPARγ, but PLIN2 is also affected by PPARα [53, 54, 55]. Meanwhile, PPARα and PPARγ regulate CIDEA expression by the peroxisome proliferator response elements in its gene promoter [56, 57]. Interestingly, CIDEA has also been reported to regulate PPARα expression [58, 59]. Despite the different models and interventions used, the relationship between PPARα and CIDEA deserves further elucidation. The direct targeting of BSCL2 by BBR also needs to be further explored.

In summary, this study provides the first preliminary investigation into the effect and mechanism of BBR on regulating LDs. BBR reduces LD size both in vivo and in vitro, which is associated with the regulation of BSCL2 and PPARα‐mediated modulation of CIDEA/PLIN4/PLIN2. These findings provide a new perspective on BBR for the treatment of NAFLD. However, it is undeniable that our study has some limitations. In vitro validation using mouse liver cell lines or primary mouse hepatocytes should be included in future work, which would greatly enhance the reliability of findings. The addition of rescue experiments to the in vivo studies could further elucidate the interaction of BBR with its targets. Moreover, further validation as well as the identification of LD‐associated proteins to which BBR can directly bind, and the pathways involved need to be refined in future work.

## 5 Conclusions

BBR exerts an active role in attenuating hepatic steatosis in DIO mice by regulating the LD‐associated proteins CIDEA, PLIN4, PLIN2 and BSCL2. In OA‐induced HepG2 cells, BBR reduced LD size by regulating BSCL2 and PPARα‐mediated CIDEA/PLIN4/PLIN2.

## Author Contributions

**Hongzhan Wang:** investigation (equal), methodology (equal), writing – original draft (lead). **Zhi Wang:** conceptualization (equal), data curation (equal), validation (equal). **Dingkun Wang:** formal analysis (equal), validation (equal). **Kexin Nie:** formal analysis (equal). **Wenbin Wu:** visualization (equal). **Yang Gao:** visualization (equal). **Shen Chen:** investigation (equal). **Xinyue Jiang:** investigation (equal). **Yueheng Tang:** methodology (equal). **Hao Su:** software (equal). **Meilin Hu:** resources (equal). **Ke Fang:** project administration (equal), resources (equal), supervision (equal). **Hui Dong:** project administration (equal), supervision (equal), writing – review and editing (lead).

## Conflicts of Interest

The authors declare no conflicts of interest.

## Supporting information


Table S1.

## Data Availability

The data that support the findings of this study are available from the corresponding author upon reasonable request.
